# Overexpression of SFPQ Improves Cognition and Memory in AD Mice

**DOI:** 10.1155/np/3934591

**Published:** 2025-01-29

**Authors:** Jinshan Tie, Hongxiang Wu, Wei Liu, Yuying Li, Lu Li, Suju Zhao, Zhijiao Yuan, Khan Mahmood, Shaochun Chen, Huidong Wu

**Affiliations:** ^1^School of Rehabilitation, Kunming Medical University, Kunming 650500, Yunnan Province, China; ^2^Rehabilitation Medicine Department, The Third People's Hospital of Yunnan Province, Kunming 650011, Yunnan Province, China

## Abstract

Alzheimer's disease (AD) is a complex neurodegenerative disorder with multifaceted pathogenesis, which has been extensively investigated, yet effective treatments remain lacking. Splicing factor proline and glutamine rich (SFPQ) is known to play a crucial role in neurodegenerative diseases, including antioxidant-related functions and regulating gene expression within brain neurons. However, the specific role of SFPQ in AD pathology is not well understood. In this study, an AD mouse model was established through lateral ventricular injection of amyloid-beta_1–42_ (A*β*_1–42_). Subsequently, adeno-associated virus was administered to overexpress SFPQ in the hippocampus of AD mice. The results demonstrate that SFPQ overexpression improves recognition and memory in AD mice, while reducing AD-related marker proteins such as amyloid precursor protein (APP) and Tau. Additionally, synaptic and memory-associated proteins, as well as antioxidant proteins like glutathione S-transferase (GST) and heme oxygenase-1 (HO-1), were upregulated. The ratio of antiapoptotic protein Bcl-2 to proapoptotic protein Bax also increased. Furthermore, phosphorylated phosphoinositide 3-kinase (p-PI3K)/PI3K and phosphorylated protein kinase B (p-AKT)/AKT ratios were elevated, indicating activation of the PI3K/AKT signaling pathway. These findings suggest that SFPQ may serve as a promising molecular target for the prevention and treatment of AD.

## 1. Introduction

With the aging of the world's population, age-related neurodegenerative diseases have become a significant unresolved issue in modern society. Over a century ago, Alzheimer's disease (AD) was identified as a degenerative disorder of the nervous system. However, during this extensive period, scientists have not developed an effective treatment to cure this complex condition. AD is the most common cause of dementia in individuals over 65 years of age. Patients with AD experience changes in memory, abstract thinking, judgment, behavior, and mood, which eventually interfere with the body's motor control. In summary, dementia places substantial burdens on patients, families, and healthcare systems.

Pathogenesis research on AD has concentrated on various hypotheses including the amyloid cascade, Tau protein, neuroinflammation, metal ions, and oxidative stress. While these hypotheses may appear independent, oxidative stress, a process that causes neuronal damage via multiple pathways [1], and serves as a unifying link between different AD hypotheses and mechanisms. Research indicates that although the brain utilizes approximately 20% of the body's oxygen intake, the high proportion of polyunsaturated fatty acids in neuronal membranes and the limited capacity of the antioxidant system render neurons particularly vulnerable to the deleterious effects of oxidative stress [2].

Numerous studies have demonstrated that A*β* peptide itself can stimulate an elevation in reactive oxygen species (ROS) both in vivo and in vitro, resulting in the manifestation of oxidative stress [3–7]. Furthermore, research indicates that the utilization of antioxidants, such as pretreatment with vitamin E and catalase, can attenuate A*β* aggregation. Autopsy findings revealed an escalation in oxidative stress products at locations of Tau aggregation in patients with AD [8]. Immunohistochemistry and immunofluorescence techniques were employed to ascertain that the end products of advanced glycation in the brains of AD patients were augmented, which can facilitate the generation of oxygen radicals and aggregates at the site of Tau protein deposition [9]. Moreover, hyperphosphorylation of Tau, microtubule disturbances, and Tau accumulation have been observed to promote ROS production [10], while oxidative stress also contributes to the formation of phosphorylated-Tau (p-Tau) and neurofibrillary tangles. In summary, oxidative stress plays a crucial role in the progression of AD by promoting A*β* deposition, Tau hyperphosphorylation, and the subsequent loss of synapses and neurons, leading to a vicious cycle. Therefore, the relationship between oxidative stress and AD suggests that oxidative stress is a significant component of the pathological process, and antioxidants may be beneficial in the treatment of AD [11, 12].

Splicing factor proline and glutamine rich (SFPQ) is a member of the Drosophila behavior human splicing (DBHS) family of RNA-binding proteins, which are crucial for various neuronal functions. This protein is highly conserved across different species and is predominantly located in the nuclei of animal cells. In healthy brain cells, SFPQ plays a vital role in regulating gene expression within neurons, helping to prevent errors in the RNA integration sequence that is essential for producing important neuronal proteins [13]. Additionally, SFPQ has been found to affect the transcriptional elongation of particularly long genes, those over 100 kilobases, in the developing murine brain. When SFPQ is absent, it can disrupt this transcriptional elongation, leading to decreased expression of these long genes and ultimately causing neuronal apoptosis [14, 15]. Many of the long genes that are downregulated are linked to neurodegenerative and psychiatric disorders, playing essential roles in processes such as axonal guidance, neuronal migration, and synaptic formation. These findings indicate that SFPQ is vital in safeguarding the regulation of gene expression, which is crucial for neuronal development and survival.

Research increasingly indicates that SFPQ plays a vital role in the development and maintenance of neurons, which is essential for their physiological function. This growing body of evidence suggests that SFPQ is significantly implicated in various neurodegenerative disorders, such as amyotrophic lateral sclerosis (ALS), frontotemporal lobar degeneration (FTLD), and AD [16–20]. SFPQ levels were found to be decreased in the postmortem brains of patients with rapidly progressing AD (rpAD), sporadic Creutzfeldt–Jakob disease (sCJD, a rapidly progressive form of dementia), and in 3xTg (amyloid precursor protein [APP]/PS1/Tau) mice. Specific knockdown of SFPQ in the hippocampus of mice leads to FTLD-like phenotypes, including p-Tau accumulation, reduced adult neurogenesis, and hippocampal atrophy with neuronal loss [21]. A subsequent investigation has revealed significant nuclear dislocation of SFPQ within the brain tissues of individuals suffering from rpAD. Additionally, there was a notable colocalization of SFPQ with p-Tau in the extranuclear space. This relationship between SFPQ and Tau oligomers found in the brains of rpAD patients suggests that SFPQ may play a crucial role in the oligomerization process and the subsequent misfolding of Tau proteins. In a study conducted with 3xTg mice, researchers observed that SFPQ expression levels were markedly increased during the early stages of the disease. Furthermore, after inducing oxidative stress in vitro, SFPQ was found within stress granules that tested positive for TIA-1. Previous studies have also indicated that inhibiting SFPQ can influence the levels of ROS in colon adenocarcinoma cells [22]. These findings suggest that SFPQ has a regulatory role in oxidative stress. Furthermore, it has been found that SFPQ knockdown promotes apoptosis in human colon cancer cells [23], indicating that SFPQ influences cell apoptosis. Therefore, it is evident that SFPQ is closely related to AD, oxidative stress, and apoptosis, and plays a crucial role in AD pathology, warranting further investigation.

In an unpublished study, our group observed a decreasing trend in SFPQ levels in the brains of mice during the aging process. Furthermore, we discovered that SFPQ levels in the hippocampus of AD mice were lower compared to age-matched control mice. However, the specific changes that occur in the hippocampus of AD mice following SFPQ overexpression and the underlying mechanisms remain unclear. To address this knowledge gap, we overexpressed SFPQ in the hippocampus of AD mice and subsequently assessed their memory and cognitive behaviors. The aim of this study was to elucidate the mechanisms by which SFPQ enhances cognitive and memory functions in AD mice. The findings of this research may provide a theoretical basis for considering SFPQ as a potential molecular target in the development of preventive and therapeutic strategies for AD.

## 2. Materials and Methods

### 2.1. Animals

This study utilized 55 specific pathogen-free (SPF) grade C57BL/6J female mice, aged 10 weeks and weighing 20 ± 2 g, procured from SPF (Beijing) Biotechnology Co., Ltd. (License number: SCXK [Beijing] 2019-0010). Ten mice served as controls, while 45 were employed for AD model. Following the adeno-associated virus (AAV) injection, three mice in the control group and three in the experimental group expired prior to the repetition of the behavioral experiments. The mice were maintained on a 12-h light/dark cycle, provided with ad libitum access to food and water, and housed at an ambient temperature of 25°C with a humidity of 45%. The study received ethical approval from the Animal Ethics Committee, Kunming Medical University, with the approval number kmmu20221753.

Cervical dislocation after isoflurane anesthesia. Anesthesia is administered at a concentration of 2% to cause the animal to lose consciousness and enter a state of deep anesthesia (the criteria for judging deep anesthesia are decreased respiratory rate, complete muscle relaxation, loss of eyelid response, and no response between the toes or between the fingers). After that, the experimental animals were euthanized with cervical dislocation.

### 2.2. Brain Stereotaxic Instrument Injection

To develop an animal model of AD, we referred to the stereotaxic atlas of the mouse brain and selected the right lateral ventricle (coordinates: anterior/posterior: −0.94 mm, medial/lateral: −1.5 mm, dorsal/ventral: −2.1 mm) for injection. We administered 5 μL of amyloid-beta_1–42_ (A*β*_1–42_) peptide at a concentration of 1 mg/mL and an injection speed of 0.2 μL/min.

### 2.3. A*β*_1–42_ Solution Preparation

To prepare the solution, 1 mL of normal saline was added to 1 mg of A*β*_1–42_ lyophilized powder (Sigma, USA). The resulting concentration of the preparation was 1 mg/mL. The solution was aliquoted into 200 μL in centrifuge tubes to prevent repeated freezing and thawing cycles.

### 2.4. AAV9-Green Fluorescent Protein (GFP) Injection

The AAV9-GFP-SFPQ and the negative control vector were synthesized by Jikai Gene Chemical Technology Co. Ltd., (Shanghai, China) for use in the experiment. The experimental group utilized an AAV-GFP system to overexpress SFPQ in the bilateral hippocampus, as guided by the stereotaxic atlas of the mouse brain (anterior/posterior: −2.3 mm; medial/lateral: ±2 mm; dorsal/ventral: ±1.75 mm). Each AD mouse received bilateral injections of 1.0 µL (virus titer: 3.4 × 10^12^ viral genomes/mL) of AAV-GFP-SFPQ in the hippocampus, while the negative control group (Vector) was administered 1.0 µL of virus without SFPQ.

### 2.5. Western Blotting

Following tissue weighing, the lysate, prepared using RIPA and PMSF at a ratio of 100:1, was added. The mixture was cracked on ice for 30 min, subjected to ultrasonic treatment, and then centrifuged in an ultra-low temperature centrifuge −4°C at 12,000 rpm for 30 min to obtain the supernatant. Protein concentration was determined using a bicinchoninic acid (BCA) assay kit (Biomed, Beijing, China). SDS-PAGE was performed, and the gel was cut according to molecular weight. The proteins on the gel were electrotransferred onto PVDF membranes and then membranes were blocked with a blocking solution for 2 h at room temperature, followed by incubation with a primary antibody at 4°C overnight. The primary antibodies used were those raised against SFPQ (Abcam, 1:10,000), APP (Proteintech, 1:1000), Tau (CST, 1:1000), GST (glutathione S-transferase) (Abcam, 1:5000), HO-1 (heme oxygenase-1) (Abcam, 1:2000), Bcl-2 (Abcam, 1:2000), Bax (Abcam, 1:2,000), PSD95 (Abcam, 1:2000), p-CREB (cAMP response element-binding) (Abcam, 1:5000), AKT (Abcam, 1:1000), phosphorylated protein kinase B (p-AKT) (Abcam, 1:1000), PI3K (Abcam, 1:1000), phosphorylated phosphoinositide 3-kinase (p-PI3K) (Abcam, 1:1000), *β*-tubulin (Abcam, 1:1000), and GAPDH (Abcam, 1:10,000) proteins. The membrane was washed three times with TBST (Tris-buffered saline with 0.1% Tween 20) for 15 min each time, followed by incubation with horseradish peroxidase (HRP)-labeled goat antirabbit/antimouse IgG (1:5000), HRP-linked antibody (CST, 1:2000) at room temperature for 2 h. The membrane was then washed three times with TBST for 15 min each time. Finally, enhanced chemiluminescence (ECL) reagents (Tanon, Shanghai, China) were used to display the protein bands, and ImageJ1.53a (National Institutes of Health, Bethesda, MD, USA) was employed to analyze the gray values of each protein band.

### 2.6. Hematoxylin and Eosin (HE) Staining

The slices were placed in the following solutions sequentially: Xylene 1 for 20 min, Xylene 2 for 20 min, absolute ethanol 1 for 5 min, absolute ethanol 2 for 5 min, and 75% alcohol for 5 min. Finally, the slices were washed with tap water. Afterward, the slices were sequentially stained with HE. The process ended with dehydration and mounting of the stained tissue samples.

### 2.7. Immunofluorescence Experiment

Frozen sections were rewarmed at room temperature for 30 min, then washed with phosphate buffer solution tween (PBST) three times, each lasting 5 min. The repair solution was heated in a microwave oven on high power for 3 min. After boiling, the slices were placed into a container and boiled on low power for 15 min, followed by natural cooling. The sections were then washed with PBST three times, each lasting 5 min, followed by a 20-min wash with 2% PBST. After washing again with PBST three times, each lasting 5 min, the tissues were circled with an immunohistochemistry pen. Ten percent goat serum was added dropwise to each tissue sample for a 2-h incubation, and a small amount of water was added to the wet box to maintain humidity. After adding a monoclonal antibody (1:50), the sections were incubated at 4°C overnight. They were then washed with PBST four times, each lasting 10 min. The secondary antibody was added, and the sections were incubated at room temperature for 2 h in the dark. Following this, they were washed with PBST four times, each lasting 10 min. Finally, the sections were sealed with a DAPI-containing sealing agent, with care taken to avoid bubbles. The slices were dried at room temperature for 4 h before images were captured using a fluorescence microscope.

### 2.8. Novel Object Recognition (NOR) Experiment

Phase 1: Adaptation period. The mice were allowed to freely explore an empty experimental setup for 10 min, serving as an initial acclimatization phase.

Phase 2: Familiarization period. Prior to the commencement of the experiment, two identical objects (A1 and A2) were secured within the apparatus, ensuring that both objects were positioned at a distance of 10 cm from the enclosing walls. Afterward, a mouse was placed into the apparatus with its back oriented toward the objects, maintaining an equal distance from each. The mouse was then allowed to explore the environment for a duration of 10 min.

Phase 3: Testing period. One hour following the completion of the familiarization period, object A1 was replaced with a novel object, denoted as object B. Subsequently, the mouse was placed into the apparatus utilizing the same methodology as described above and allowed to explore the environment for a duration of 10 min.

During each stage, a camera and specialized software were utilized to record the mouse's trajectory and the time spent exploring each object. The RI was calculated as follows: RI = (Time exploring new object B)/(Time exploring new object B + time exploring familiar object A2) × 100%.

### 2.9. Y-Maze Test

The Y-maze consisted of three arms: the start arm, the novel arm, and the familiar arm. The experiment was conducted in two stages. In Stage 1, the novel arm was closed, and a mouse was placed in the start arm, allowing it to explore the maze for 5 min. After a 1-h interval, Stage 2 commenced. The novel arm was opened, and the mouse was again placed in the start arm. The mouse's activity was observed for 5 min. The proportion of time the mouse spent in the novel arm relative to the total time spent in all three arms was analyzed.

### 2.10. Statistical Analysis

Statistical analysis was performed using R statistical software (version 4.2.1, R Foundation for Statistical Computing). Tests for normality and homogeneity of variance were conducted to determine the appropriate statistical tests. The independent *t*-test was employed for data meeting both normality and homogeneity of variance assumptions, while Welch's *t*-test was utilized for data satisfying the normality condition but not the homogeneity of variance assumption. Two-tailed *p* values less than 0.05 were considered statistically significant.

## 3. Results

### 3.1. Successful Establishment of the AD Model and Improved Memory and Cognition in AD Mice Following SFPQ Overexpression

**Figure 1:**
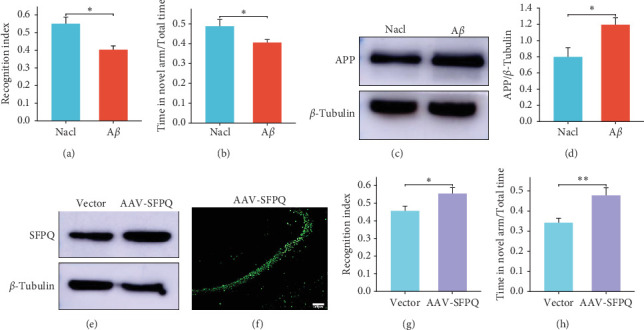
AD mice were successfully modeled, and SFPQ was overexpressed in the hippocampus. (A, B) The NOR and Y-maze experiments demonstrated successful AD modeling (NaCl group, *n* = 7; A*β* group, *n* = 42). (C, D) Western blotting and quantitative analysis revealed increased APP levels in the hippocampus of AD mice, confirming successful modeling. (E, F) Western blotting and immunofluorescence showed SFPQ overexpression in the hippocampus (scale bar = 100 μm). (G) Following SFPQ overexpression, AD mice exhibited improved performance in the novel object recognition task. The Vector group was commonly used for comparison with the AAV group. *⁣*^*∗*^*p* < 0.05. (H) SFPQ overexpression enhanced AD mice performance in the Y-maze test. *⁣*^*∗*^*⁣*^*∗*^*p* < 0.01. AD, Alzheimer's disease; SFPQ, splicing factor proline and glutamine rich.

In this study, the NaCl group, serving as the control, received an injection of 5 µL of normal saline, while the A*β* group was injected with 5 µL of A*β*_1–42_ into the lateral ventricles. After 14 days, the model was successfully verified through behavioral experiments, including the NOR and Y-maze tests (Figure 1A,B), with a 100% survival rate among the mice. The results of WB showed that the APP content in the AAV-SFPQ group decreased (Figure 1C,D). Subsequently, AAV expressing SFPQ (AAV-SFPQ) was injected into the hippocampus of the AD mice. Western blotting analysis revealed a higher expression of SFPQ in the hippocampus of the AAV-SFPQ group compared to the Vector group (Figure 1E). As AAV-SFPQ expresses GFP, fluorescence microscopy was utilized to confirm the overexpression of SFPQ in the hippocampus (Figure 1F). Four weeks postinjection, behavioral experiments were repeated, demonstrating that AD mice injected with AAV-SFPQ exhibited enhanced cognitive performance in the NOR and Y-maze tests compared to the control group, indicating that SFPQ overexpression improves cognition and memory in AD mice (Figure 1G,H).

### 3.2. Overexpressed SFPQ Colocalized With CA1 Neurons and Improved the Arrangement of Hippocampal Cells in AD Mice

**Figure 2:**
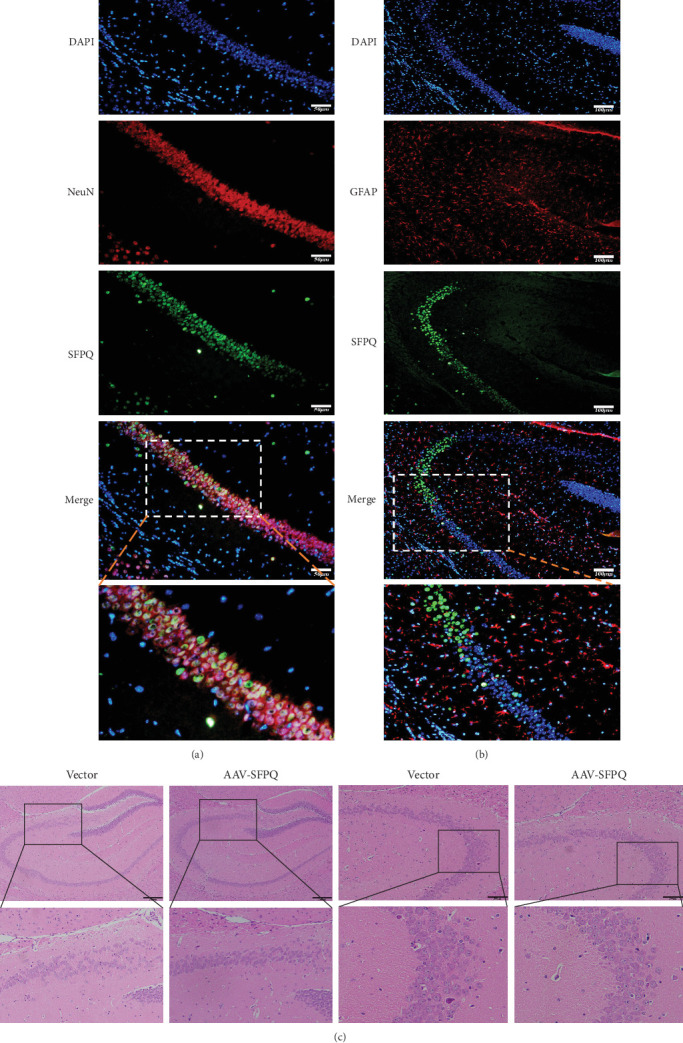
SFPQ colocalized with neurons in the hippocampal CA1 region and arrangement of hippocampal cells improved after SFPQ overexpression. (A) Immunofluorescence demonstrated that the overexpressed SFPQ primarily colocalized with neurons in the CA1 region. Scale bar = 100 μm. (B) SFPQ did not colocalize with astrocytes. Scale bar = 50 μm. (C) HE staining revealed morphological differences in the hippocampal cells, particularly in the CA3 and CA4 regions, between the Vector and AAV-SFPQ groups. Scale bar = 200 μm. SFPQ, splicing factor proline and glutamine rich.

To determine whether SFPQ was overexpressed in neurons or astrocytes, immunofluorescence colocalization analysis was conducted using the neuronal marker NeuN and the astrocyte marker GFAP. The results demonstrated that SFPQ primarily colocalized with neurons in the CA1 region (Figure 2A), while no significant colocalization was observed between SFPQ and astrocytes (Figure 2B).

To investigate potential morphological changes in the hippocampus of AD mice following SFPQ overexpression, HE staining was conducted. HE staining results revealed that, compared to the Vector group, the hippocampal cells in the CA3 and CA4 regions of the AAV-SFPQ group exhibited a more organized, compact, uniform, and morphologically intact arrangement. In contrast, the cells in the CA3 and CA4 regions of the Vector group appeared disorganized, sparse, and irregular (Figure 2C). HE staining provides insight into the general tissue structure, including the nucleus, cytoplasm, and overall morphology of various cell and tissue types. A disorganized arrangement of the CA3 region in the hippocampus typically indicates a pathological signal, suggesting potential damage or dysfunction in this area. Previous studies have demonstrated that lesions in the hippocampus, particularly in the CA3 region, are closely associated with the onset of AD, potentially leading to neuronal disorganization, cell loss, synaptic dysfunction, and consequent memory impairment [24–26]. These findings suggest that SFPQ overexpression improved the hippocampal arrangement in AD mice, providing a possible explanation for the observed behavioral enhancements.

### 3.3. Overexpression of SFPQ Downregulated AD-Related Marker Proteins and Upregulated the Expression of Memory and Synapse-Associated Proteins

**Figure 3:**
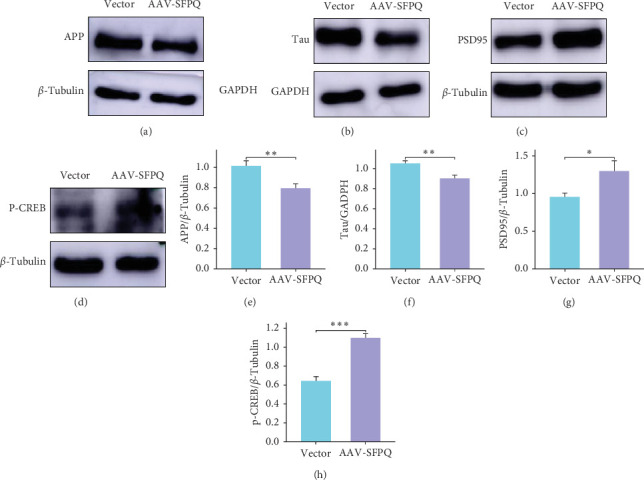
AD-related and memory-associated proteins have changed. (A, B) After overexpression of SFPQ, the expression of AD-related proteins APP and Tau decreased. (C, D) The expression of synaptic and memory-related proteins PSD95 and p-CREB increased in the AAV-SFPQ group. (E–H) Quantification analysis of APP, Tau, PSD95, and p-CREB. *n* = 9, data were expressed as mean ± standard error of the mean (SEM). *⁣*^*∗*^*p* < 0.05,  ^*∗*^^*∗*^*p* < 0.01,  ^*∗*^^*∗*^*p* < 0.001. AAV, adeno-associated virus; AD, Alzheimer's disease; CREB, cAMP response element-binding; SFPQ, splicing factor proline and glutamine rich.

Compared to the Vector group, the expression of APP in the AAV-SFPQ group exhibited a significant decrease (Figure 3A). Concurrently, Tau protein levels also demonstrated a downward trend (Figure 3B). These findings suggest that the overexpression of SFPQ may potentially alleviate AD symptoms through the downregulation of APP and Tau expression.

When brain nerve cells are active, CREB protein can activate and contribute to the formation of long-term memory. Since SFPQ was found to improve the memory of AD mice, we next determined if phosphorylated-CREB (p-CREB) protein level was increased in the AAV-SFPQ group. As revealed by western blotting results, the expression of p-CREB was elevated in the AAV-SFPQ group (Figure 3D), indicating CREB activation. Additionally, the expression of postsynaptic density protein 95 (PSD95), another memory-related protein, was also elevated in the AAV-SFPQ group (Figure 3C). PSD95 is a component of the postsynaptic membrane in the nervous system that is involved in the formation of synaptic connections, which further facilitates memory improvement. Therefore, SFPQ can enhance cognition and memory in AD mice by activating CREB and increasing synaptic connections.

### 3.4. Overexpression of SFPQ Enhanced Antioxidant and Antiapoptotic Capacities in Hippocampal Cells of the AD Mouse Model

**Figure 4:**
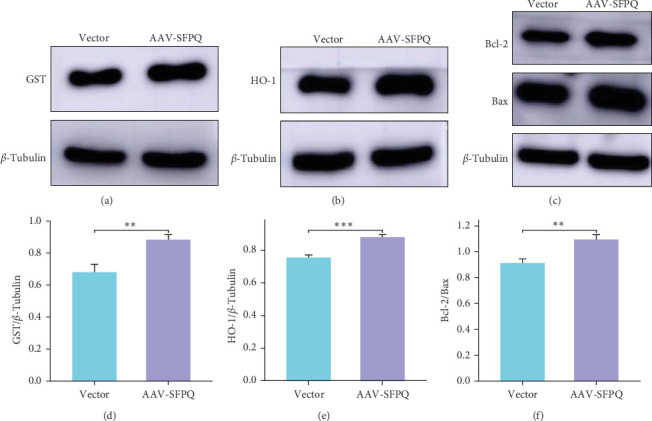
Overexpression of SFPQ resulted in increased levels of antioxidant and antiapoptotic proteins. (A, B) The expression of the antioxidant proteins GST and HO-1. (C) The expression of the antiapoptotic proteins Bcl-2/Bax. (D–F) Quantification analysis of GST, HO-1, and Bcl-2/Bax. *n* = 9, data were expressed as mean ± SEM.  ^*∗*^^*∗*^*p* < 0.01,  ^*∗*^^*∗*^*p* < 0.001. GST, glutathione S-transferase; SEM, standard error of the mean; SFPQ, splicing factor proline and glutamine rich.

Previous studies have associated SFPQ with antioxidants, which may be related to behavioral improvements following SFPQ overexpression. To investigate this connection, we measured antioxidant protein levels. Compared to the Vector group, the AAV-SFPQ group exhibited enhanced antioxidant capacity in the hippocampus, with significantly increased levels of GST and HO-1 (Figure 4A,B). Additionally, the AAV-SFPQ group demonstrated improved antiapoptotic ability relative to the Vector group. Although both Bcl-2 and Bax increased in the AAV-SFPQ group, the ratio of Bcl-2/Bax increased, and the difference was statistically significant (Figure 4C). These findings suggest that SFPQ overexpression enhances the antioxidant and antiapoptotic capabilities of hippocampal neurons in AD mice, effectively protecting hippocampal neuronal function and improving cognitive and memory abilities in these animals.

### 3.5. Overexpression of SFPQ Activated the PI3K/AKT Pathway

**Figure 5:**
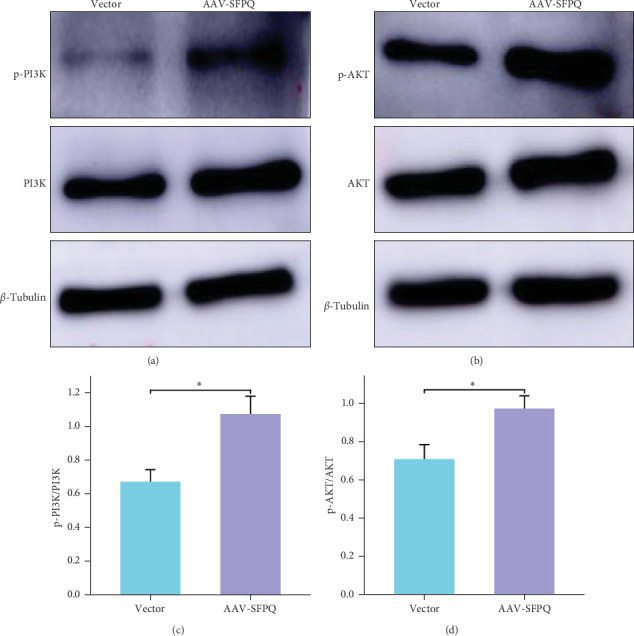
Key proteins in the PI3K/AKT pathway were increased. (A, B) Following SFPQ overexpression, the ratios of p-PI3K/PI3K and p-AKT/AKT were elevated. (C, D) Quantification analysis of p-PI3K/PI3K and p-AKT/AKT ratios, indicating activation of the PI3K/AKT pathway. *n* = 9, data were expressed as mean ± SEM. *⁣*^*∗*^*p* < 0.05. SEM, standard error of the mean; SFPQ, splicing factor proline and glutamine rich.

The PI3K pathway plays a crucial role in maintaining synaptic plasticity in the adult brain and significantly impacts memory processes. The PI3K/AKT pathway's antiapoptotic and prosurvival effects underscore its importance in AD. Furthermore, SFPQ has demonstrated antioxidant and antiapoptotic properties in other diseases, suggesting a potential intrinsic relationship between SFPQ and the PI3K/AKT pathway. Compared to the Vector group, the SFPQ overexpression group exhibited increased expression of PI3K, p-PI3K, and enhanced p-PI3K/PI3K ratio (Figure 5A). Additionally, the expressions of AKT, P-AKT, and p-AKT/AKT ratio were elevated (Figure 5B). These findings indicate that the PI3K/AKT pathway is activated following the overexpression of SFPQ.

## 4. Discussion

Our previous research results demonstrated that SFPQ levels increased following oxidative stress and decreased in the hippocampus of aged mice. These findings suggest that SFPQ may play an antioxidant role in AD mouse models, although the specific mechanism remains unclear. This study explored the potential mechanisms underlying the behavioral improvements in AD mice following SFPQ overexpression. After 4 weeks of SFPQ overexpression in the hippocampus of AD mice, we observed significant improvements in both the NOR and Y-maze tests, indicating enhanced memory and cognitive function. These findings highlight the crucial role of SFPQ in AD pathology and its potential as a therapeutic target.

Individuals with dementia frequently experience difficulty in forming new memories. Research has demonstrated that the formation of new memories involves DNA double-strand breaks and a 24-h repair process [27]. However, in diseased states, the DNA repair process is compromised. Evidence suggests that SFPQ plays a critical role in repairing DNA double-strand breaks [28]. The dislocation of SFPQ may contribute to defects in DNA repair, which subsequently impacts the formation of new memories. While this study does not confirm the dislocation of SFPQ, its overexpression may enhance DNA repair and consequently improve cognitive function in mice with AD.

With advancing age, the brain encounters challenges in maintaining redox balance, resulting in the accumulation of ROS that disrupt crucial cellular pathways and contribute to cell aging and death. This oxidative damage is widely acknowledged as a prodromal feature associated with neurodegeneration in AD. The present study demonstrated that overexpressing SFPQ in the hippocampus of AD mice significantly elevated the levels of antioxidant enzymes GST and HO-1. GST, an antioxidant enzyme, exerts its antioxidant effects through various pathways. HO-1, a key nuclear factor erythroid 2-related factor 2 (NRF2) target gene, possesses the ability to reverse oxidative damage and stress, with its upregulation being regarded as a cellular defense mechanism against oxidative stress. These findings suggest that SFPQ overexpression could increase antioxidant enzyme levels, enhance antioxidant function, and mitigate the oxidative damage induced by A*β*.

Patients with AD commonly exhibit decreased executive function and reasoning, which result from changes in the synaptic proteome [29, 30]. In addition, synaptic loss in neurodegenerative diseases is often associated with the accumulation of neurotoxic substances. Research has shown that A*β*_42_ oligomers can cause oxidative damage to synaptic membranes [31, 32], leading to defects in long-term potentiation. PSD95 is an indicator of the postsynaptic membrane. It is also a crucial component of glutamatergic transmission, synaptic plasticity, and dendritic spine morphogenesis during neurodevelopment [33]. Decreased expression of PSD95, which is involved in synapse formation and reconstruction, implies a loss of synaptic function [34, 35]. This study confirmed that overexpression of SFPQ in AD mice increased PSD95 expression. This suggests that SFPQ may help to restore synaptic function, providing an alternative explanation for the observed improvements in cognition and memory in these mice. Taken together, SFPQ may have decreased damage to synaptic membranes and lowered synaptic loss by alleviating oxidative stress and reducing A*β* deposition in AD mice.

Tau protein plays a critical role as a pathological factor in AD. SFPQ acts as a splicing factor that suppresses 4R-Tau expression by selectively splicing Tau exon 10. By modulating the 4R/3R-Tau ratio, SFPQ may also promote Tau aggregation and phosphorylation in Tau-related disorders [36]. Studies have demonstrated that mice exhibit frontotemporal dementia-like behavior following the knockout of SFPQ in the hippocampus. These mice also underwent various cellular and molecular alterations, including reduced adult neurogenesis, neuronal loss in the hippocampus, and the accumulation of p-Tau protein [21]. Although this study did not explore the specific function of SFPQ as a splicing factor, the observed decrease in Tau following SFPQ overexpression may be associated with its splicing function.

This study investigated the presence and functionality of overexpressed SFPQ protein in various hippocampal cell types, as SFPQ is expressed in both neurons and astrocytes. Immunofluorescence colocalization revealed that overexpressed SFPQ primarily resides in neurons rather than astrocytes. This may be attributed to the predominant impact of oxidative damage on neurons, while astrocytes exhibit higher resistance, supporting the antioxidant role of SFPQ. Moreover, the primary pathological changes in AD occur in neurons, where overexpressed SFPQ is also found, resulting in improved hippocampal neuron morphology. Overexpressed SFPQ is more frequently observed in neurons of the CA1 region, potentially due to the distinct cell types, quantities, and functions in different hippocampal regions. CA1 region neurons possess prominent signaling pathways crucial for long-term memory and spatial tasks, although the underlying mechanisms require further investigation. However, the specific mechanisms underlying these effects warrant further exploration.

This study demonstrates that SFPQ overexpression activates the PI3K/AKT signaling pathway. According to existing research, the PI3K/AKT signaling pathway serves multiple functions, including inhibiting A*β*-induced apoptosis, activating *α*-secretase to promote the production of nonamyloidogenic APP, and regulating the expression of mitochondrial membrane permeability proteins such as Bcl-2 and Bax. Furthermore, the PI3K pathway is crucial for maintaining synaptic plasticity in the adult brain and plays a significant role in memory processes [37]. Since our study did not directly demonstrate that SFPQ's antiapoptotic and antioxidant effects were mediated through the PI3K/AKT pathway, further evidence is required in future studies to establish this relationship conclusively.

The multifaceted etiology of AD and the intricate interactions among various pathological factors necessitate a comprehensive therapeutic approach with multiple functions. As a result, comprehensive therapy is gaining attention as an emerging strategy for treating AD. Given the beneficial effects of SFPQ toward AD mice, this protein may be developed into an effective molecular target in the prevention and treatment of this disease.

## 5. Conclusions

In conclusion, the cognition and memory of AD mice were significantly improved following the overexpression of SFPQ, indicating that SFPQ plays a crucial and beneficial role in murine cognitive function. These findings establish a theoretical foundation for the potential use of SFPQ as a therapeutic target in AD treatment. Future research is necessary to elucidate the specific mechanisms through which SFPQ exerts its effects in AD pathology and to assess its suitability for clinical application.

## 6. Limitations

This study conducted behavioral experiments and evaluated expression changes in AD-related marker proteins, apoptosis, and antioxidant proteins in animal models after SFPQ overexpression; however, it did not thoroughly investigate the regulatory relationships or interactions among these proteins. Although changes in PI3K/AKT pathway proteins following SFPQ overexpression were observed, it remains to be demonstrated whether SFPQ overexpression exerts its antiapoptotic and antioxidative effects solely through the PI3K/AKT pathway.

## Data Availability

Supporting data are available from the corresponding author upon reasonable request.
